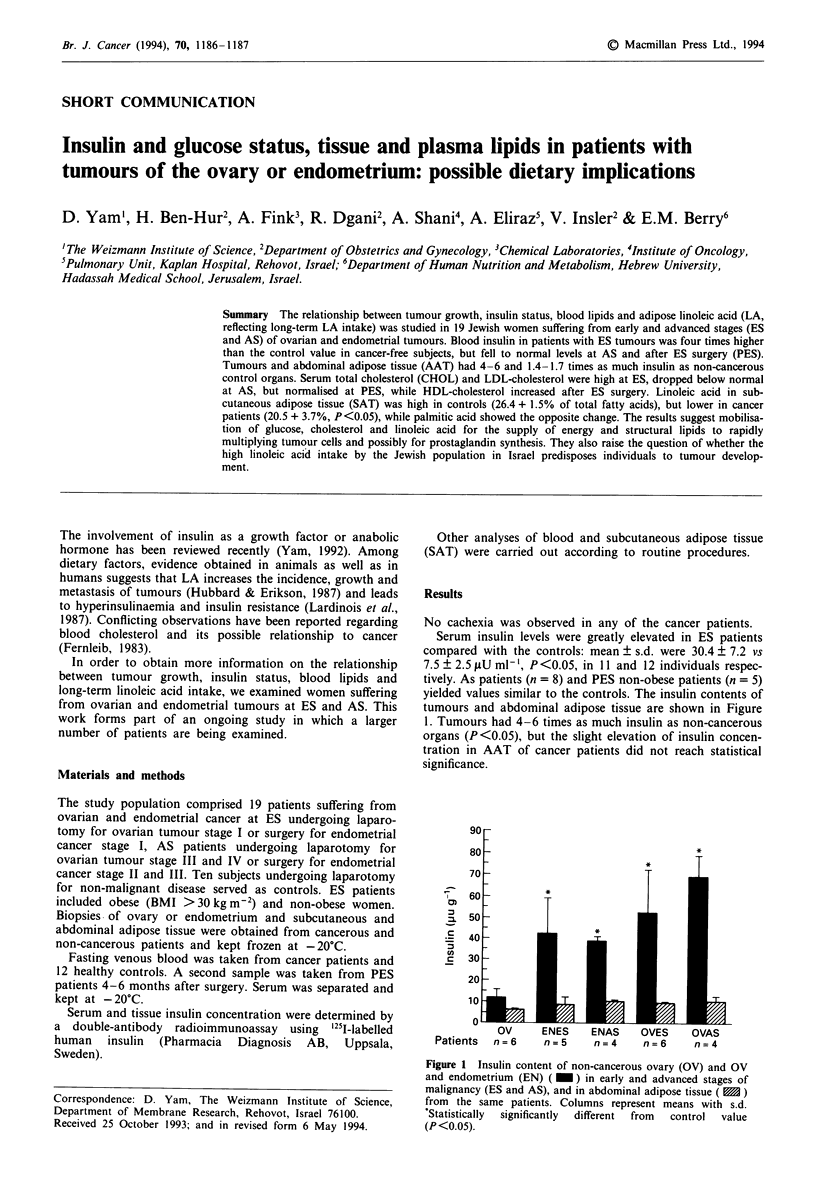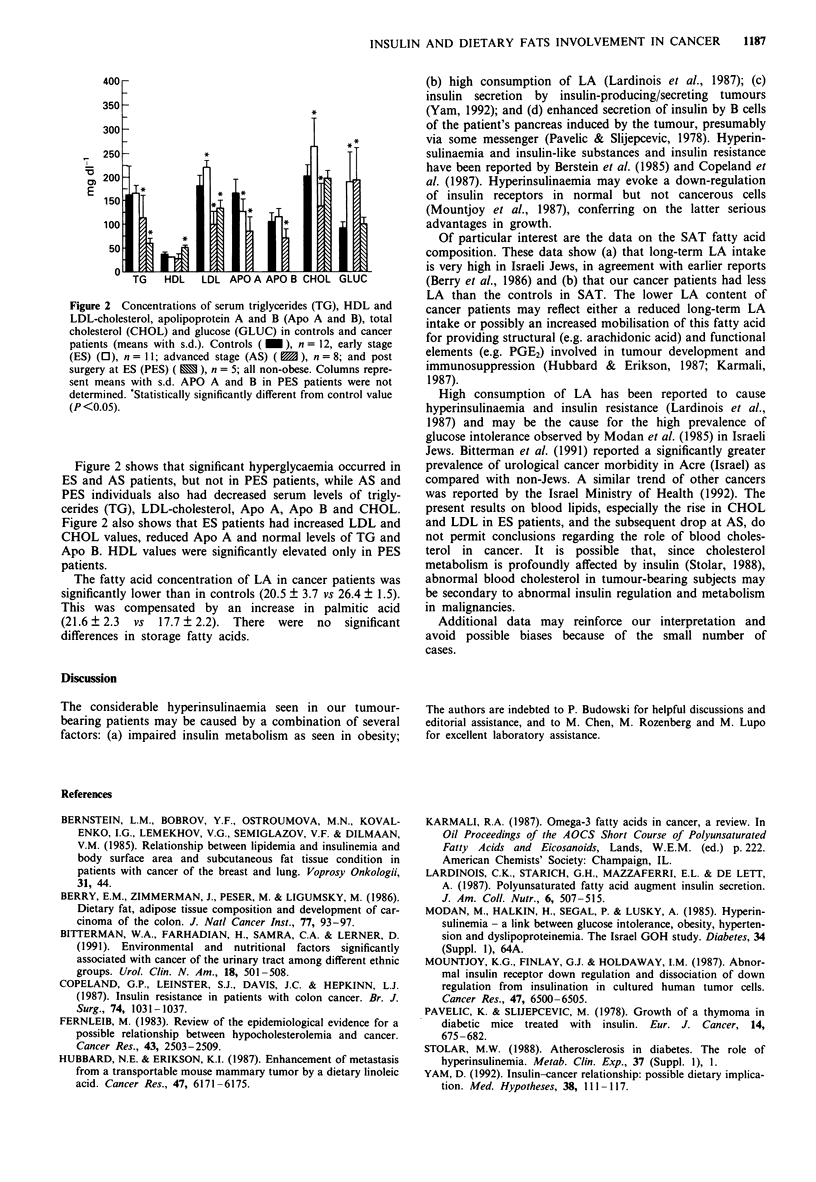# Insulin and glucose status, tissue and plasma lipids in patients with tumours of the ovary or endometrium: possible dietary implications.

**DOI:** 10.1038/bjc.1994.470

**Published:** 1994-12

**Authors:** D. Yam, H. Ben-Hur, A. Fink, R. Dgani, A. Shani, A. Eliraz, V. Insler, E. M. Berry

**Affiliations:** Weizmann Institute of Science, Rehovot, Israel.

## Abstract

The relationship between tumour growth, insulin status, blood lipids and adipose linoleic acid (LA, reflecting long-term LA intake) was studied in 19 Jewish women suffering from early and advanced stages (ES and AS) of ovarian and endometrial tumours. Blood insulin in patients with ES tumours was four times higher than the control value in cancer-free subjects, but fell to normal levels at AS and after ES surgery (PES). Tumours and abdominal adipose tissue (AAT) had 4-6 and 1.4-1.7 times as much insulin as non-cancerous control organs. Serum total cholesterol (CHOL) and LDL-cholesterol were high at ES, dropped below normal at AS, but normalised at PES, while HDL-cholesterol increased after ES surgery. Linoleic acid in subcutaneous adipose tissue (SAT) was high in controls (26.4 + 1.5% of total fatty acids), but lower in cancer patients (20.5 + 3.7%, P < 0.05), while palmitic acid showed the opposite change. The results suggest mobilisation of glucose, cholesterol and linoleic acid for the supply of energy and structural lipids to rapidly multiplying tumour cells and possibly for prostaglandin synthesis. They also raise the question of whether the high linoleic acid intake by the Jewish population in Israel predisposes individuals to tumour development.


*Br. J. Cancer* (1994), **70**, 1186–1187 © Macmillan Press Ltd., 1994

SHORT COMMUNICATION

### Insulin and glucose status, tissue and plasma lipids in patients with tumours of the ovary or endometrium: possible dietary implications

D. Yam[1], H. Ben-Hur[2], A. Fink[3], R. Dgani[2], A. Shani[4], A. Eliraz[5], V. Insler[2] & E.M. Berry[6]

[1]*The Weizmann Institute of Science,* [2]*Department of Obstetrics and Gynecology,* [3]*Chemical Laboratories,* [4]*Institute of Oncology,* [5]*Pulmonary Unit, Kaplan Hospital, Rehovot, Israel;* [6]*Department of Human Nutrition and Metabolism, Hebrew University, Hadassah Medical School, Jerusalem, Israel.*

**Summary** The relationship between tumour growth, insulin status, blood lipids and adipose linoleic acid (LA, reflecting long-term LA intake) was studied in 19 Jewish women suffering from early and advanced stages (ES and AS) of ovarian and endometrial tumours. Blood insulin in patients with ES tumours was four times higher than the control value in cancer-free subjects, but fell to normal levels at AS and after ES surgery (PES). Tumours and abdominal adipose tissue (AAT) had 4–6 and 1.4–1.7 times as much insulin as non-cancerous control organs. Serum total cholesterol (CHOL) and LDL-cholesterol were high at ES, dropped below normal at AS, but normalised at PES, while HDL-cholesterol increased after ES surgery. Linoleic acid in subcutaneous adipose tissue (SAT) was high in controls (26.4 + 1.5% of total fatty acids), but lower in cancer patients (20.5 + 3.7%, $P<0.05$), while palmitic acid showed the opposite change. The results suggest mobilisation of glucose, cholesterol and linoleic acid for the supply of energy and structural lipids to rapidly multiplying tumour cells and possibly for prostaglandin synthesis. They also raise the question of whether the high linoleic acid intake by the Jewish population in Israel predisposes individuals to tumour development.

The involvement of insulin as a growth factor or anabolic hormone has been reviewed recently (Yam, 1992). Among dietary factors, evidence obtained in animals as well as in humans suggests that LA increases the incidence, growth and metastasis of tumours (Hubbard & Erikson, 1987) and leads to hyperinsulinaemia and insulin resistance (Lardinois *et al.*, 1987). Conflicting observations have been reported regarding blood cholesterol and its possible relationship to cancer (Fernleib, 1983).

In order to obtain more information on the relationship between tumour growth, insulin status, blood lipids and long-term linoleic acid intake, we examined women suffering from ovarian and endometrial tumours at ES and AS. This work forms part of an ongoing study in which a larger number of patients are being examined.

### Materials and methods

The study population comprised 19 patients suffering from ovarian and endometrial cancer at ES undergoing laparotomy for ovarian tumour stage I or surgery for endometrial cancer stage I, AS patients undergoing laparotomy for ovarian tumour stage III and IV or surgery for endometrial cancer stage II and III. Ten subjects undergoing laparotomy for non-malignant disease served as controls. ES patients included obese (BMI $>30\,kg\,m^{-2}$) and non-obese women. Biopsies of ovary or endometrium and subcutaneous and abdominal adipose tissue were obtained from cancerous and non-cancerous patients and kept frozen at $-20°C$.

Fasting venous blood was taken from cancer patients and 12 healthy controls. A second sample was taken from PES patients 4–6 months after surgery. Serum was separated and kept at $-20°C$.

Serum and tissue insulin concentration were determined by a double-antibody radioimmunoassay using $^{125}$I-labelled human insulin (Pharmacia Diagnosis AB, Uppsala, Sweden).

Correspondence: D. Yam, The Weizmann Institute of Science, Department of Membrane Research, Rehovot, Israel 76100.
Received 25 October 1993; and in revised form 6 May 1994.

Other analyses of blood and subcutaneous adipose tissue (SAT) were carried out according to routine procedures.

### Results

No cachexia was observed in any of the cancer patients.

Serum insulin levels were greatly elevated in ES patients compared with the controls: mean ± s.d. were 30.4 ± 7.2 *vs* 7.5 ± 2.5 $\mu U\,ml^{-1}$, $P<0.05$, in 11 and 12 individuals respectively. As patients ($n$ = 8) and PES non-obese patients ($n$ = 5) yielded values similar to the controls. The insulin contents of tumours and abdominal adipose tissue are shown in Figure 1. Tumours had 4–6 times as much insulin as non-cancerous organs ($P<0.05$), but the slight elevation of insulin concentration in AAT of cancer patients did not reach statistical significance.

**Figure 1** Insulin content of non-cancerous ovary (OV) and OV and endometrium (EN) (■) in early and advanced stages of malignancy (ES and AS), and in abdominal adipose tissue (▨) from the same patients. Columns represent means with s.d. *Statistically significantly different from control value ($P<0.05$).

**Figure 2** Concentrations of serum triglycerides (TG), HDL and LDL-cholesterol, apolipoprotein A and B (Apo A and B), total cholesterol (CHOL) and glucose (GLUC) in controls and cancer patients (means with s.d.). Controls (■), $n = 12$, early stage (ES) (□), $n = 11$; advanced stage (AS) (▨), $n = 8$; and post surgery at ES (PES) (▧), $n = 5$; all non-obese. Columns represent means with s.d. APO A and B in PES patients were not determined. *Statistically significantly different from control value ($P<0.05$).

Figure 2 shows that significant hyperglycaemia occurred in ES and AS patients, but not in PES patients, while AS and PES individuals also had decreased serum levels of triglycerides (TG), LDL-cholesterol, Apo A, Apo B and CHOL. Figure 2 also shows that ES patients had increased LDL and CHOL values, reduced Apo A and normal levels of TG and Apo B. HDL values were significantly elevated only in PES patients.

The fatty acid concentration of LA in cancer patients was significantly lower than in controls (20.5 ± 3.7 *vs* 26.4 ± 1.5). This was compensated by an increase in palmitic acid (21.6 ± 2.3 *vs* 17.7 ± 2.2). There were no significant differences in storage fatty acids.

### Discussion

The considerable hyperinsulinaemia seen in our tumour-bearing patients may be caused by a combination of several factors: (a) impaired insulin metabolism as seen in obesity; (b) high consumption of LA (Lardinois *et al.*, 1987); (c) insulin secretion by insulin-producing/secreting tumours (Yam, 1992); and (d) enhanced secretion of insulin by B cells of the patient's pancreas induced by the tumour, presumably via some messenger (Pavelic & Slijepcevic, 1978). Hyperinsulinaemia and insulin-like substances and insulin resistance have been reported by Berstein *et al.* (1985) and Copeland *et al.* (1987). Hyperinsulinaemia may evoke a down-regulation of insulin receptors in normal but not cancerous cells (Mountjoy *et al.*, 1987), conferring on the latter serious advantages in growth.

Of particular interest are the data on the SAT fatty acid composition. These data show (a) that long-term LA intake is very high in Israeli Jews, in agreement with earlier reports (Berry *et al.*, 1986) and (b) that our cancer patients had less LA than the controls in SAT. The lower LA content of cancer patients may reflect either a reduced long-term LA intake or possibly an increased mobilisation of this fatty acid for providing structural (e.g. arachidonic acid) and functional elements (e.g. $PGE_2$) involved in tumour development and immunosuppression (Hubbard & Erikson, 1987; Karmali, 1987).

High consumption of LA has been reported to cause hyperinsulinaemia and insulin resistance (Lardinois *et al.*, 1987) and may be the cause for the high prevalence of glucose intolerance observed by Modan *et al.* (1985) in Israeli Jews. Bitterman *et al.* (1991) reported a significantly greater prevalence of urological cancer morbidity in Acre (Israel) as compared with non-Jews. A similar trend of other cancers was reported by the Israel Ministry of Health (1992). The present results on blood lipids, especially the rise in CHOL and LDL in ES patients, and the subsequent drop at AS, do not permit conclusions regarding the role of blood cholesterol in cancer. It is possible that, since cholesterol metabolism is profoundly affected by insulin (Stolar, 1988), abnormal blood cholesterol in tumour-bearing subjects may be secondary to abnormal insulin regulation and metabolism in malignancies.

Additional data may reinforce our interpretation and avoid possible biases because of the small number of cases.

The authors are indebted to P. Budowski for helpful discussions and editorial assistance, and to M. Chen, M. Rozenberg and M. Lupo for excellent laboratory assistance.

### References

BERNSTEIN, L.M., BOBROV, Y.F., OSTROUMOVA, M.N., KOVALENKO, I.G., LEMEKHOV, V.G., SEMIGLAZOV, V.F. & DILMAAN, V.M. (1985). Relationship between lipidemia and insulinemia and body surface area and subcutaneous fat tissue condition in patients with cancer of the breast and lung. *Voprosy Onkologii*, **31**, 44.

BERRY, E.M., ZIMMERMAN, J., PESER, M. & LIGUMSKY, M. (1986). Dietary fat, adipose tissue composition and development of carcinoma of the colon. *J. Natl Cancer Inst.*, **77**, 93–97.

BITTERMAN, W.A., FARHADIAN, H., SAMRA, C.A. & LERNER, D. (1991). Environmental and nutritional factors significantly associated with cancer of the urinary tract among different ethnic groups. *Urol. Clin. N. Am.*, **18**, 501–508.

COPELAND, G.P., LEINSTER, S.J., DAVIS, J.C. & HEPKINN, L.J. (1987). Insulin resistance in patients with colon cancer. *Br. J. Surg.*, **74**, 1031–1037.

FERNLEIB, M. (1983). Review of the epidemiological evidence for a possible relationship between hypocholesterolemia and cancer. *Cancer Res.*, **43**, 2503–2509.

HUBBARD, N.E. & ERIKSON, K.I. (1987). Enhancement of metastasis from a transportable mouse mammary tumor by a dietary linoleic acid. *Cancer Res.*, **47**, 6171–6175.

KARMALI, R.A. (1987). Omega-3 fatty acids in cancer, a review. In *Oil Proceedings of the AOCS Short Course of Polyunsaturated Fatty Acids and Eicosanoids*, Lands, W.E.M. (ed.) p. 222. American Chemists' Society: Champaign, IL.

LARDINOIS, C.K., STARICH, G.H., MAZZAFERRI, E.L. & DE LETT, A. (1987). Polyunsaturated fatty acid augment insulin secretion. *J. Am. Coll. Nutr.*, **6**, 507–515.

MODAN, M., HALKIN, H., SEGAL, P. & LUSKY, A. (1985). Hyperinsulinemia – a link between glucose intolerance, obesity, hypertension and dyslipoproteinemia. The Israel GOH study. *Diabetes*, **34** (Suppl. 1), 64A.

MOUNTJOY, K.G., FINLAY, G.J. & HOLDAWAY, I.M. (1987). Abnormal insulin receptor down regulation and dissociation of down regulation from insulination in cultured human tumor cells. *Cancer Res.*, **47**, 6500–6505.

PAVELIC, K. & SLIJEPCEVIC, M. (1978). Growth of a thymoma in diabetic mice treated with insulin. *Eur. J. Cancer*, **14**, 675–682.

STOLAR, M.W. (1988). Atherosclerosis in diabetes. The role of hyperinsulinemia. *Metab. Clin. Exp.*, **37** (Suppl. 1), 1.

YAM, D. (1992). Insulin–cancer relationship: possible dietary implication. *Med. Hypotheses*, **38**, 111–117.